# Strategies for Aging in Place: The Experience of Language-Minority Seniors With Loss of Independence

**DOI:** 10.1177/2333393614565187

**Published:** 2015-02-04

**Authors:** Suzanne Dupuis-Blanchard, Odette N. Gould, Caroline Gibbons, Majella Simard, Sophie Éthier, Lita Villalon

**Affiliations:** 1 Université de Moncton, Moncton, New Brunswick, Canada; 2 Mount Allison University, Sackville, New Brunswick, Canada; 3 Université Laval, Quebec, Canada

**Keywords:** older people, families, community and public health, descriptive methods, language, long-term health care

## Abstract

For healthy and independent older adults, aging in place can be seen as identical to any other adult living at home. Little is known about how frail seniors, particularly those who speak a minority language, manage the challenges of aging in place. The present qualitative descriptive study explores the strategies that Canadian French-speaking seniors have put in place to counter their loss of independence and promote their ability to stay in their home. Semistructured individual interviews were conducted with 39 older adults and transcribed, followed by content analysis to identify common themes related to study objectives. Six themes emerged in response to strategies described for aging in place. Findings reveal the limited extent to which language issues were perceived as a barrier by participants. In conclusion, the results of this study provide us with fruitful insights to guide community nursing practice, future research, and public policy.

Population aging is a global trend because of longer life expectancy and lower fertility rates (World Health Organization, 2013) and is reflected in many demographic predictions. For example, it is projected that by the year 2015, 22% of the world population will be above the age of 60, and most adults in developed countries will live well into their 80s and 90s. In Canada, it is predicted that by 2015, older adults will outnumber youths (Canadian Institute for Health Information, 2011), and by the year 2036, one in four citizens will be a senior (Statistics Canada, 2013).

One of the concerns related to population aging is the issue of institutionalization of older adults, both in terms of economic costs for the country and quality of life for institutionalized older adults. In Canada, 92% of adults above the age of 65 live at home (Statistics Canada, 2013), whereas 8% live in institutions. Although the majority of older adults are able to remain at home, the absence of home care in the *Canada Health Act* leads to difficulties accessing services and to long waiting lists for many older adults who require care (Canadian Home Care Association, 2008). Moreover, factors such as speaking a minority language, having a low income, not having family members close by, and requiring extra hours of home care can make aging in place difficult (Dupuis-Blanchard, Simard, Gould, & Villalon, 2013). In fact, these factors (in addition to the multiple challenges of home maintenance, transportation, and activities of daily living) can deter older adults from staying at home.

In Canada, 31% of the population can speak French. Despite the *Official Languages Act* in Canada, French-speaking older adults have difficulties accessing home support services in their language (Bouchard, 2012). In fact, some older adults forego receiving services in French to more quickly access needed services. However, for some French-speaking older adults in the province of New Brunswick, accepting services in English proves difficult because of the language barrier. As a result, language is a social determinant of health, with an impact on health services use and health status (Bouchard, 2012). For French-speaking older adults residing in mostly English-speaking communities, most services are not offered in their language of choice. This reality dissuades French-speaking older adults and their families to seek services and care for aging at home.

The province of New Brunswick offers an ideal location to study aging in place in minority groups. Approximately 33% of the residents of the province report French as their first language, 43% say that they can have a conversation in French (Statistics Canada, 2013), and minority-group members are distributed across both rural and urban areas. Importantly, the provinces of Atlantic Canada boast the fastest aging population in Canada (Statistics Canada, 2013), provoking concerns about the costs of providing health- and long-term care in the future. Currently, older adults in New Brunswick who can no longer stay at home are often hospitalized for an average of 6 months (New Brunswick Department of Social Services, personal communication, June 24, 2014) while awaiting admission to a long-term care facility. Clearly, this approach to managing the loss of independence of our aging population is not viable economically. Moreover, there are suggestions that these high societal costs are leading to a public backlash against these frail persons, as the term “bedblocker” is often used to describe this population. Strategies to increase the number of seniors who can live at home despite their loss of independence are therefore important to consider because they may offer solutions to alleviate demands on institutional long-term care.

Aging in place is rooted in the ecological model of aging (Lawton & Nahemow, 1973), in which the capabilities of the person to meet the demands of the environment result in successful interaction. In the context of aging in place, when the interplay of people and environment proves to be a challenge, older adults want to modify their home to stay independent.

Aging in place is the preference of many older adults (Ballard et al., 2011; Cutchin, 2003; Dupuis-Blanchard, Neufeld, & Strang, 2009). Defined as living in one’s own home in the community (American Association of Retired Persons [AARP], 2005), aging in place involves being able to identify resources to support personal and environmental change (Fänge, Oswald, & Clemson, 2012) and respond to essential needs. Community resources include home-maintenance services such as lawn care and housework, or home care such as health monitoring and nursing care (Canadian Home Care Association, 2008). These services are offered in most communities, but often are not sufficient to meet the demands as a result of financial cutbacks and the unavailability of human resources (Grenier & Guberman, 2009).

Within the context of aging in place, *home* can be defined as a detached house, a condominium, or an apartment unit, including apartments in senior-designated buildings. In fact, only since the 1990s has the term *aging in place* become popular among older adults, family members, health care professionals, and policy makers. Indeed, researchers and policy makers in particular have focused their interest on non-community-residing seniors rather than on the majority of seniors who are aging “normally” (Peace, Holland, & Kellaher, 2011).

There are many advantages to aging in place. Economically, providing help with home maintenance or home care is dramatically less expensive than institutional care (Canadian Institute for Health Information, 2008). Moreover, the social and health advantages of aging at home have been extensively documented (Cutchin, 2003; Joosten, 2007; Wagnild, 2001) and include attachment to place, familiarity with the neighborhood, and the ability to maintain functional health.

For healthy and independent older adults, aging in place can be seen as identical to any other adult living at home. However, little is known about how frail seniors, particularly those who speak a minority language, manage the challenges of aging in place in a context in which services are often refused because of language. The present article explores the strategies that these seniors have put in place to counter their loss of independence and promote their ability to stay in their home, despite the challenges of accessing services in their language. Because studies have shown that home support services and transportation are inadequate (Cannuscio, Block, & Kawachi, 2003; Dupuis-Blanchard, Simard, Gould, & Villalon, 2011, 2013; Fausset, Kelly, Rogers, & Fisk, 2011), it is imperative to better understand how French-speaking older adults living with a loss of independence can age in place. The goal of the present study was to gain a better understanding of aging in place by exploring how older adults who experience a loss of independence and speak a minority language (French) are able to remain in their homes, despite the multiple challenges they face.

## Method

Because extensive prior research was lacking in this specific area of aging in place, a qualitative descriptive design (Sandelowski, 2000) was used to understand the strategies developed to allow for aging in place by French-speaking older adults experiencing a loss of independence. Qualitative research was ideal because it is designed to provide a deep understanding of how people experience their lives (Morse & Field, 1995).

### Participants

Participants in this study were 39 older adults residing at home but experiencing a loss of independence, as well as 14 family members. Ten family members were relatives of older participants in the study, and four were relatives of an older person with loss of independence who resided at home.

### Data Collection and Analysis

The study was conducted from March 2012 through June 2013. After university ethics approval, recruitment of older participants was initiated by posters, announcements in various community bulletins, word of mouth using community gatekeepers, and presentations at community events. To facilitate recruitment of older adults with loss of independence, recruiting materials stated that we were seeking older adults who required assistance to accomplish some of their daily activities. All participants self-identified as French speaking (francophone). Participants were recruited in four separate communities: rural francophone and anglophone communities, and urban francophone and anglophone communities. Family members were recruited by leaving a letter of invitation with older participants and posting advertisements throughout the community. With both older participants and family members, data collection consisted of a semistructured individual interview in the participants’ homes that lasted an average of 90 min. None of the older adults resided with the family member interviewed. With participant consent, interviews were audiotaped and transcribed verbatim for analysis. Guiding questions encouraged older participants to explain how they managed to stay in their homes, despite the possible language barrier and the loss of independence; interviews with family members examined their experience with an older relative aging in place. During interviews of family members with an older adult in the study, no information was shared regarding the content of the interview with the older adult. At the end of each interview, participants were asked sociodemographic information. Data saturation was achieved for both types of participants when no new information emerged from the interviews.

Verbatim transcriptions were analyzed line by line using open coding, and with each new interview, clusters of data were examined for similarities and differences, and then collected into larger clusters labeled *categories*. Categories that explained participants’ beliefs and behaviors were grouped into larger categories. This sifting process (Morse, 1994) left only the important features of data relevant to the study. Participants were not solicited to participate in the analysis, nor did they review their transcripts. Also, analysis was conducted separately for the older adult group and the family members’ group. In this study, data analysis was facilitated by the use of NVivo 8 software by QSR International. Coders were native French speakers of the same ethnic origin as the participants. The research team was comprised of a nurse, a psychologist, a nutritionist, a social worker, and a geographer, all with previous experience working together on other projects related to aging in place. All French quotes presented in the results section were translated from French to English by bilingual coders.

## Results

### Sample

The average age of the older participants was 81 years, with a range of 65 to 93 years. In all, 22 older participants were female, 11 participants were married, 24 were widowed, 2 were divorced, and 2 were never married. The majority of older adults had less than a high school education, and most had a yearly income of $50,000 (CAD) or less. Twenty-one older adults in this study self-evaluated their health as good, and the most common health problems were hypertension and mobility limitations. Nineteen older adults still drove a car, and half of the older participants resided in a rural community. The majority of family members were in their 50s, and most had a college or a university education. Eight family members were women, and six were male. All participants in this study were Caucasian.

Six themes emerged in response to the strategies older participants defined as vital for aging in place. These strategies are defined as personal- and community-level themes that allow older adults to age at home. Themes include (a) attitude and self-determination, (b) health consciousness, (c) housing choice, (d) access to services, (e) social support, and (f) income.

### Attitude and Self-Determination

A positive attitude was described by older participants as essential for aging in place. Such an attitude was defined as being optimistic about the future and believing that one can adapt to any circumstances. Although many participants mentioned the difficulties of staying in their home, the advantages seemed to outweigh any challenges. Many of the advantages mentioned by both older participants and family members included quality of life, tranquility, independence, and socializing. Nearly all participants were adamant about staying in their homes for as long as possible. “Well, it’s a house for living in; everything is handy. I’ll never go, I tell you.”

Self-determination was also an important element of autonomy and aging in place, and was defined as doing what is needed to stay independent. Older adults and family members explained the importance of keeping active and doing for oneself what must be done:

I was putting my garbage out at the back door; it’s steep, and the lady next door could see me and she asked me if I wanted her to take that out for me. I said no, I have to do this.

Another older participant explained, “Keep going from one day to the next. That’s all I can do. Do whatever I am able to do. I force myself to keep going.” This attitude of self-determination brought some older participants to ask for help to stay in their home longer, whereas others expressed difficulties and reluctance in seeking help. A daughter explained,

To be honest, I really don’t know how much longer he is going to stay in his own home. He may want it, but I can’t see it, unless he agrees and he’s willing to accept that somebody comes into the house.

Health problems seemed to influence older adults’ self-determination. These health challenges brought many older participants to make changes to their lifestyle and social outings. However, their positive attitude prevailed, and most believed that they would regain their optimum level of health over time. Even family members explained, “Even if she can’t prepare her meals anymore, I can live with that. I’ll organize something.”

### Health Consciousness

Older participants related good health to the ability of aging in place; therefore, lifestyle, daily health routines, and taking charge of one’s health were described as important. Being physically active, having nutritious meals, and a healthy activity/rest ratio were described by most participants. One older participant explained, “I keep active and I try not to eat things I’m not supposed to. As long as I keep moving, the pain and arthritis doesn’t bother me as much.” Another older participant stated,

I have a stationary bike. I’m on that every morning after I get out of bed, before I do anything else, before I even take my pajamas off, I’m on the bike. I don’t go for long, maybe four or five minutes, but it’s a good habit. I fear if I don’t do that, my heart will just get weaker and my lungs will also get weaker, and eventually I’ll just drop dead.

A son worried about meals:

I worry about their meals. Her appetite is quite small. For a while last year, she wasn’t eating at all. She stopped functioning and she got so weak, we had to put her in the hospital for three days. Now she’s pretty good.

A daily routine was also deemed important. Preparing meals, grocery shopping, cleaning, and managing finances were described as essential components of aging in place. Although described as elements of aging in place, a number of older participants verbalized, “It’s boring to do meals every day. It’s no fun to cook for one. I can do it, but I don’t enjoy it.” Others explained, “I don’t make all of my meals. I visit friends and I have invitations out.” Grocery shopping can also present its challenges:

I go in and get the groceries. I mean, you’re holding on to the cart, so there’s no problem. It’s just that it takes me a longer time and instead of a half an hour, well, it’s an hour and a half or two hours.

One older participant described how difficult it is to let go of certain daily routines, although conscious of the health benefits of being active:

It’s hard because you sit here all day and you think “I should be dusting or doing this and that,” but I can’t do it because I just start and then I’m beat. It’s pretty dusty most of the time.

Another explained how she has adapted her personal routine: “We just have a standard bathtub, and I have a seat that goes in it and the bars. I never get in unless my husband is here.”

The practice of favorite activities was described as a means to lessen loneliness and the activities themselves were social in nature. Ranging from singing in a choir to playing Bingo, most agreed that it is an essential component of successfully aging in place. Leaving home for these activities proves challenging for some with mobility issues. As explained by an older participant’s son,

He has a walker but refuses to use it! He was doing very well with it but as soon as he got home, he put the walker away. He didn’t want anybody seeing him using a walker. You can go more places if you use the walker, but he wouldn’t think of it that way. It was all about how he looked to other people.

With time, some older participants realized the benefits of mobility aids. A son explained, “Mother uses a cane and she has been very resistant, but in the last six months or so, she’s more comfortable with it and now she has a sense of being independent.”

### Housing Choice

Older participants explained that they presently lived in a home where their needs were met. Some participants who lived in a two-story house explained that they had closed off the second story and now lived in the main floor: “My bedroom and washroom are on the first floor. I don’t use the second floor anymore.” One woman was thinking of making changes: “My washer and dryer are downstairs now and I’m thinking if I’m going to stay here, I’m going to have it brought up to the main level.” Others explained that when they had relocated in the past, they had chosen a home with few stairs and wide doorways:

We lived in our own home. It was two levels with six steps to the lower level and 13 steps to the second floor. We just decided we can’t do this anymore. We sold the house and came to an apartment.

Another participant shared, “One of the things I looked for in this home was the wide concept, to make it accessible if I ever had to be in a wheelchair.”

Outdoor maintenance and cleaning were presented by many of the older participants as challenging tasks. Lawn mowing, snow clearing, painting, and washing floors, walls, and windows were identified as challenges to aging in place. Other participants explained that they had decided to downsize and relocate to an apartment. Without the worries of outdoor maintenance, apartments are attractive housing options for older adults who are no longer able or willing to take care of larger living spaces. Many, including family members, attributed apartment living to feelings of security: “We’d feel better if he was in a place where there were people around. At least if people haven’t seen him in a day or so, they’d check on him.” For other older participants, living in an apartment unit was construed as a loss of privacy. One older participant living in her large house said, “I like the freedom. You don’t have neighbors upstairs driving you crazy, or next door, or downstairs. I enjoy the quiet, the peace, and I guess the part of knowing that I have my own house; it’s paid for.”

Many expressed opinions about other types of housing. For instance, older participants living in a house shared their views of living in an apartment unit: “In an apartment, you’re not at home. You can’t paint and you can have a building manager that does not allow for any social activities.” Others expressed concern over living in a long-term care facility: “You have to get up at a certain time, eat when they tell you, and go to bed early. I hope I die before going into a nursing home.”

### Access to Services

A variety of services may be needed to age in place. One service that was mentioned by most participants was access to transportation. Owning a car seemed to facilitate aging in place and access to community resources, health care, and social activities. Besides its usefulness, owning a car is symbolic of autonomy and independence. As one older participant explained, “If there’s something I want, I just jump in the car and go and get it. I almost surprised myself at the age of 80 when I bought a brand-new car.” However, many had modified their driving habits: “I don’t drive when the sun goes down and at night.”

Older participants who live in urban areas also have access to public transportation, but not without problems. “Well, the bus didn’t come within a mile to where she lived so she couldn’t get the groceries. There is a bus system, but it only goes here and there.” Some participants explained that they use taxi services to get to appointments, but that it is very costly: “Some months, it cost me $150 for cabs to run to the hospital and to the doctor.” For participants living in rural communities, access to transportation is very problematic. “Some sort of public transportation would be a big help. But we tried that a while ago and it didn’t last very long. Not enough people for a bus system, and a taxi service would be too expensive.” Many rural residents form networks and share a drive to the grocery store or elsewhere.

Older adults who can no longer drive or do not possess a car rely mostly on family and friends: “I don’t have a car and that is the most difficult. I have to depend on my children. That’s inconvenient and I find it difficult.” Although grocery shopping, medical appointments, going to church, and social activities are important to older participants, many are reluctant to ask for a drive:

I gave up driving four years ago. A neighbor will take me. Oh, I’d try this neighbor first, but I know these days, I don’t try on Tuesdays and Thursdays because he’s busy. If I’m really stuck, another neighbor will take me. I have a good idea of their schedules; you get on to it.

Older participants also explained that access to other services is also important. Such services include delivery of hot meals, house cleaning, and health care services in one’s language. One rural older participant explained, “It’s nice here, but it’s far away from the doctors, the dentist, and other specialists.” Some also shared their frustrations about home care services:

They weren’t consistent with coming. They came for three hours, two times per week. A half hour of that was spent on break, a half hour of that was spent on telling somebody new what you needed help with; so it left you two hours to maybe go get my groceries and do my house stuff. I had to pay their way in a cab to go wherever I need to go. And then it just got crazy.

### Social Support

Aging in place would not be possible for older participants if it were not for social support, the formal and informal help provided by family members and neighbors. For some, the presence of family members, including spouses, was indispensable for staying at home. Support from family members included preparing meals, housework, transportation, and accompaniment during medical appointments. Neighbors also provided support, especially in rural areas:

I have a friend who lives next door, she’s 39 and she does a lot of nice things for me. She’s a hairstylist, but she’s also a bank manager. She cuts my hair and she won’t let me pay her. She treats me like a parent.

Despite the availability of support networks, many older participants are hesitant to ask family members for help: “If I really need anything done, I could get one of my daughters-in-law to come, but they all work and have their own families and their own homes. You hate to ask them all the time to do things.” Instead of asking family members, some have developed support networks with others their age:

My mom will get on the phone and call up somebody in the building who is also old, but who has offered help to get to the grocery store. Mother is more and more open to take advantage of that; she doesn’t feel guilty that she’s imposing on somebody.

As explained by an older lady, things are just not the same:

I don’t go out like I used to because it’s too difficult. I belong to a lunch group, I do keep it up; I go with another lady who comes and picks me up at the door. There are lots of things that I’d like to do that I can’t do, like go shopping.

Despite the strong desire to stay at home, older participants shared their concerns relating to isolation and loneliness: “It’s difficult being alone so much. It’s depressing at times, but I manage. Weekends are the worst. That’s when I hate being alone.” Others described ways to cope with being alone: “I go visit people. I have several friends around that I visit, that I can walk to.” Other older participants use technology: “I use the computer quite a bit. I used to go the library, but I can’t anymore, so I use the computer. I play on it; check emails; read a book.” Family members also shared other similar thoughts:

My mom’s a very sociable person, loves to have people around. That is probably what I see as her biggest challenge of living alone and not having that social aspect that she’s used to. I would love to see my mom move to an environment where there are other people, like a seniors’ apartment complex.

The community is also a source of support for older participants. Through social inclusion, access to community resources, communication, and public environments that promote independence, older adults are allowed to age in their respective communities. Rural communities seemed to offer a feeling of belonging to older participants.

A son shared this: “She loves to go to the bank because three or four of the staff members, as soon as she walks in, they say hello. She has this personal connection with them; that’s a really big thing for her.” Older participants commented on the importance of having services available in their community: “We’re in a very fortunate area. The bank, the liquor store, the grocery store; they’re all right there. The pharmacy is across the street.” However, in rural areas, the lack of available services in the community creates a great challenge to aging in place. In some communities, the grocery store, bank, and gas station have closed, and all that remain are the church and a community center with few scheduled activities. Older adults who remain independent often volunteer to drive other seniors to the nearby community for grocery shopping and banking.

### Income

Older participants shared that adequate income and education contribute to facilitate aging in place. Those participants in good financial health were able to pay for private services such as housekeeping, transportation, and general house maintenance. An older participant explained,

With myself, I can hire somebody. Like we had the roof done last year and we had a chimney taken down, and the whole side of the house done, and a piece of ceiling because we had water coming through. We hired a carpenter to come and do it, but a lot of people can’t do that.

Those seniors who could hire someone for help often commented during the interviews about other older adults who could not afford private services and did not qualify for publicly subsidized help. Lower income participants explained that they would make choices on how to spend their money, and some explained that they would limit their social outings to be able to pay for a taxi for grocery shopping:

There are all kinds of things I used to do that I can’t do anymore. I was in a lot of things, but now I don’t have any money to do things. I can’t go to the theatre anymore because I can’t afford to. And you can’t volunteer because there’s no extra money to pay your way to get there.

Education was also mentioned by older participants as an important factor in staying independent. Most participants associated education level with being able to identify resources in the community. Although many years had passed since older participants’ school days, participants shared many experiences of being bullied because of their French culture. Most participants explained that they were obligated to learn the English language to go to school and find employment.

## Discussion

Six strategies were identified by study participants as contributing to successful aging in place. Personal characteristics such as positive attitude and self-determination, being health conscious, and having adequate income were recognized as contributing to aging in place; community-based dimensions included housing options, access to services, and social support. Although living with a loss of independence, older adults in this study mobilized their resources and put strategies in place to allow them to age in place for as long as possible. Interviews with family members also revealed that they provide the much needed support to their aging family members.

The ability to adapt to changing needs is essential for aging in place (Fänge et al., 2012). In fact, participants in our study who identified themselves as aging well had the most positive outlook on life compared with those who identified with a lack of well-being, also found by Hammarström and Torres (2012). In addition, as in our study, aging in place provided feelings of belonging, security, familiarity, and independence (Wiles, Leibing, Guberman, Reeve, & Allen, 2012), while providing a place for family members to interact (Luborsky, Lysack, & Van Nuil, 2011). Likewise, older study participants in our study residing in rural communities had been long-term rural residents and had a feeling of belonging to the community (Winterton & Warburton, 2012).

Difficulties with transportation were identified by most participants in this study. In fact, older minority-group women and older adults with low income and education have the most barriers to transportation (Kim, 2011). For older participants in this study, transportation is synonymous with independence. Transportation to medical appointments, grocery shopping, and similar errands is recognized as essential by most, but outings for social activities are not. Family members and neighbors are more than willing to help out an older adult when the outing is deemed essential, but social activities are often left aside. As in past research, the majority of older adults in this study who can no longer drive seek assistance from a friend or a family member (Kim, 2011) and believe that they can rely on neighbors for help (Norstrand, Glicksman, Lubben, & Kleban, 2012).

Most of the inconveniences identified with aging in place were related to household chores. In fact, in a study by Fausset et al. (2011), 70% of participants identified difficulties with cleaning and outdoor-related tasks. Although few inconveniences were identified to living at home in the present study, it is interesting to note that participants living in single-family homes had negative beliefs about other living arrangements such as apartment living or long-term care facilities. By the same token, older participants living in apartment units were adamant that it was the best housing option for all seniors, and some stated that they wished they had left their house for an apartment much earlier.

One particular characteristic of the home discussed as a barrier to aging in place was the presence of stairs. Older adults identified challenges to using stairs to get to the second floor or the basement. Stairs have proven to be difficult for other older adults (Liu, Chang, & Huang, 2012) and one of the primary reasons for modifying the home’s inside environment (Luborsky et al., 2011). Older adults have improved chances of remaining at home after renovations have been completed (Safran-Northon, 2010).

Social support is also a required component of aging in place. Family members repeatedly stated that they provided various amounts of formal and informal support to their aging family members to allow them to age at home. Likewise, older participants recognized the contribution of family members but also stated their desire for a structured routine. Such routines allow both older adults and family members to schedule certain times for specific tasks without having to ask or confirm (Birnholtz & Jones-Rounds, 2010). In rural communities, there is a naturally occurring support system that can develop, thus following the notion of naturally occurring retirement communities (NORCs; Kloseck, Grilly, & Gutman, 2010; Sim, Liddle, Bernard, Scharf, & Bartlam, 2012).

One of the most surprising findings of this study was the limited extent to which language issues were perceived as a barrier by these older adults. Despite the fact that accessing services in the minority language in the province of New Brunswick is challenging (Forgues, Doucet, & Guignard, 2011), the older adults in this study rarely discussed language barriers. One acknowledged reason for this is that participants often depended on family members and friends to help them when they had to interact with physicians, especially specialists who did not speak French. Also, many did not receive services by choice, and language may have contributed to their decision. This generation of French-speaking residents of the province, in particular, might have not been sufficiently politicized to highlight language as a barrier. This issue deserves more attention in future research.

Nurses and other health care professionals providing care to minority-language older adults in the community need to consider the strategies identified in this study for aging in place. Communication is the basis of a safe practice, and efforts must be made to offer services in the client’s language. Additional studies are needed to better understand the realities of accessing services and receiving care for minority-language seniors. When assessing health of an older client with loss of independence in a community setting, nurses must include questions about transportation issues, attendance at social activities, grocery shopping and meal preparation, loneliness, and home safety. Availability of social support and having sufficient income are also important determinants of aging in place.

The interviews carried out provided a fascinating glimpse into the experiences of these frail seniors with loss of independence and speaking a minority language. However, the project did not proceed without difficulties. In particular, the recruitment of older participants proved to be a challenge. As a team, we have extensive experience with community-based research, but recruiting older adults with a loss of independence was difficult. Surprisingly, presentations at gatherings for older adults in our target communities did not enable us to recruit any participants. One possible explanation is that none of the potential participants wanted to identify themselves as living with a loss of independence in front of other older adults, even when the project coordinator was sensitive to this issue in the language used to describe the inclusion criteria for the study. In the end, it was only with the assistance of community gatekeepers that we were able to recruit older participants. The gatekeepers used their knowledge of community residents to distribute information letters about the study to potential study participants, who then contacted us directly. We experienced the same situation in each of the four communities in this study. Of course, it is possible that this recruiting strategy led to a biased sample. It should also be noted that only seniors who were successful at aging in place were included in this project. Further research needs to explore the experiences and decision-making processes of the older adults who chose (or are forced) to move into an institution.

A participant characteristic not portrayed in our study but believed to be important is the required components for older adults with dementia to age in place. None of the participants in this current study demonstrated or verbalized a diagnosis of dementia. With the growing prevalence of dementia in older adults, future studies should include participants living at home with a diagnosis of dementia (Fänge et al., 2012). Future studies are also needed to better understand the phenomenon of aging in place of older adults living in official language-minority communities and culturally diverse populations to inform systems of care (Ballard et al., 2011). Also, studies with other minority-language older adults on the issue of aging in place and access to services would be beneficial. In addition, this study began to explore the processes involved in asking for and accepting help from others. Further studies are needed to better understand the complex patterns of beliefs and attitudes that underlie what it means for an older adult who is losing autonomy to ask for help.

## Conclusion

The results of this qualitative study provide us with potentially fruitful insights to guide nursing practice, future research, and public policy. It seems clear that being poor, being a non-driver, and not having access to helpful neighbors and family members are all barriers that interact to hinder successful aging in place. Although this study does not allow us to draw strong conclusions in regard to minority-language status, further studies on this issue are warranted. The particular insight that this project yields, however, is the importance of the interactive nature of these barriers: Strengths in one area can make up for weaknesses in others. For example, an older adult who has the financial means to pay for certain services can make up for not being a driver and not having an informal support network. By the same token, many of our participants were very resilient in the face of quite extreme levels of poverty through the support of neighbors and family members, particularly if they had access to transportation. This notion of resiliency in the face of barriers clearly deserves research attention.

Finally, we propose that our data also suggest that service providers need to use a more bottom-up approach in planning and implementing services. Much attention has been paid in recent years to educate older adults about the value of staying active and maintaining a good diet. Our results suggest that this happens quite “naturally” when older adults are aging in place comfortably. Perhaps resources also need to be used to provide services (e.g., transportation to social events, help with yard care, and support for informal caregivers) that seniors and their family members do not consider “essential” for survival, but may be essential for a quality of life that encourages and allows aging in place.
